# The impact of Corona pandemic on consumer's food consumption: Vulnerability of households with children and income losses and change in sustainable consumption behavior

**DOI:** 10.1007/s00003-021-01341-1

**Published:** 2021-08-16

**Authors:** Adriano Profeta, Shahida Anusha Siddiqui, Sergiy Smetana, Sayed Mahdi Hossaini, Volker Heinz, Christian Kircher

**Affiliations:** DIL e.V.-German Institute of Food Technologies, Prof.-von-Klitzing-Straße 7, 49610 Quakenbrück, Germany

**Keywords:** Covid-19, Corona, Consumer behaviour, Food, Health

## Abstract

The ongoing corona crisis affected many people worldwide by restrictions in their everyday lives. The question arises to what extent the pandemic has accelerated diet trends or general differences in food consumption between different population groups. For this purpose, an online-survey was carried out in order to determine the effects of the corona lockdown on food consumption, shopping behaviour and eating habits in Germany. The aspects of sustainability and health were given special consideration in this study, reflecting people choices of healthier and more environmentally conscious foods. This study demonstrates that the corona pandemic has a significant impact on consumers’ eating habits. More food was eaten, and more convenience products such as ready-made meals and canned food with a longer shelf life were purchased. The consumption of alcohol and sweets has also increased. In return, there was a reduced consumption of fresh fruits and vegetables. The findings reveal that families who are financially affected by the pandemic represent a vulnerable group. With the ongoing pandemic, possible lockdowns, corona-related closings of schools and kindergartens, severe health consequences are expected long term, especially for this population group.

**Supplementary information:**

The online version contains supplementary material available at 10.1007/s00003-021-01341-1.

## Introduction

The ongoing corona pandemic affects many people worldwide by restrictions in their everyday lives. It is to highlight that the pandemic has also influenced the eating behaviour and shopping habits of consumers (Scarpa and Rose 2008). Due to possible quarantine phases, consumers were concerned about which type of food and its quantities should be stored (Dammeyer 2020). In addition, there were short-term out-of-stock situations in the food retail sector (Liu et al. 2020) for selected products (e.g. flour, pasta, disinfectants, etc.).

In this context, the Lower Saxony State Food Industry Association—LI Food^1^ carried out a representative online survey with 973 participants in April 2020, during the first lockdown in Germany. The survey aimed to determine the effects of the corona pandemic on food consumption, shopping behaviour and eating habits in Germany. The aspects of sustainability and health were given special consideration in the study, reflecting people choices of healthier and more environmentally conscious foods. Moreover, the survey reflected whether there were changes in the consumption quantity in different product categories or food waste avoidance. Changes in purchases of organic and locally produced products due to corona effects were also considered. The focus was primarily on households with children and households affected by a loss of income due to the lockdown. In general, the question arises to what extent the pandemic has accelerated diet trends or general differences in food consumption between different population groups. The next section briefly describes existing studies on the subject before presenting the methodology and the study results.

### Psychological and physical effects of the pandemic and possible effects on the eating and purchasing behaviour of consumers

#### “More food - an instrument for reducing stress”

It is to hypothesize that the pandemic has directly impacted people’s psyche. Even in regions with a relatively low risk of infection, the population was exposed to massive risk communication and media reporting in 2020, which in itself was a relevant psychological stress factor. Also, a considerable part of the population was affected by short-time work or unemployment or was worried about a possible job loss, which could likewise impact psychological well-being (Monsivais et al. 2015; van Hal 2015). In such situation, eating “more” can be a coping strategy to polarize ones’ psychology in order to deal with the pandemic and the stress it causes. Increased consumption of (alcoholic) beverages and food can represent an attempt to feel better under stress temporarily (Conway et al. 1981; Laitinen et al. 2002). For Italy’s first lockdown, it was observed that households mainly consumed more processed “comfort foods” such as chocolate, chips and snacks (Bracale and Vaccaro 2020; Scarmozzino and Visioli 2020). The longer the lockdown measures persists, the higher is the probability that a permanent risen consumption leads to a higher prevalence of obesity. The consequences could be a rise in diabetes, coronary diseases and cancer in the population (Swinburn et al. 2019).

#### Reduced physical activity as an amplifying factor for negative effects

During the pandemic, physical and sport activities in the population have declined. Children and parents alike spent more time with computer games and online media (Spitzer 2020). In the current edition of the specialist journal “Nervenheilkunde”, an article by the well-known neurologist Spitzer (2020) anecdotally quotes a teacher as follows:

“The students, whom I was able to see again for a few hours, spent the 7 weeks almost without exception playing in front of the computer. On average they were 5–10 kilos heavier [...]”.

The fatal consequences of the outlined situation becomes more obvious with the background of a publication in Lancet, that states that a 10% reduction in physical inactivity could lead to additional 533,000 deaths per year worldwide (Wen and Wu 2012).

#### Fear as a driver for the (increased) purchase of specific product categories

Another mediator of consumer behaviour is the associated risk with Covid-19 of falling ill or dying. Consumers could reduce their risk of infection by using delivery services or buying more packaged foods that are considered more hygienic (Bracale and Vaccaro 2020). It can also be assumed that foods with a longer shelf life will be bought (and thus fewer fresh products such as fruits and vegetables) to minimize shopping frequency and risk of infection in supermarkets. Monteiro (2009) and Monteiro et al. (2011) argue that more processed foods have a negative impact on consumers’ health status. Contrarily, there is the behavioural strategy of buying healthier food to strengthen the immune system (Rodríguez-Pérez et al. 2020). It is conceivable that this could result in an increasing demand for more fruits and vegetables or ecologically and regionally produced food.

Another factor influencing consumer behaviour are concerns about food shortages, leading to certain food stockpiling (Bracale and Vaccaro 2020). The market research institute Innofact (Düsseldorf, Germany) interviewed 1037 consumers from March 24th to 25th, 2020, and found that every third German bought significantly more noodles, ready-made meals, toilet paper, rice, flour, and kitchen rolls.^2^ This behaviour change is confirmed by scanner data from the Federal Statistical Office for calendar weeks 9–16, in which there was a “shopping boom” for the products mentioned. The overview across some recent study results, show a trend towards increased consumption and towards stockpiling.

#### Consequences of corona-related income loss or unemployment

In Germany, the pandemic led to increased unemployment and short-time work due to the pandemic.^3^ Besides, there is evidence that with lower education levels, the proportion of people who went on short-time work, unpaid vacation or unemployment was higher. A job loss is twice as likely for someone with an intermediate level of education than for someone with a high level of education. In addition, it was mainly employees with a higher education who had the opportunity to work from home. As a result, this group was exposed to a significantly lower risk of infection than those in employment with an intermediate or low level of education (Naumann et al. 2020).

A study from the UK (Hendrickson et al. 2006; Monsivais et al. 2015) showed that losing a job can lead to weight gain over certain time. Furthermore, in low-income households, the (high) price can be an obstacle to buying fruit and vegetables (Cassady et al. 2007). During the financial crisis, spending on groceries were reduced in many western industrialized countries, which can be ascribed to a decline in income (Antentas and Vivas 2014; Vlontzos and Duquenne 2013). Since the corona pandemic also represents an economic crisis, it is to hypothesize that the effects described can also be transferred to the recent situation.

## Materials and methods

A population of 973 consumers was interviewed via an online survey about their eating, buying and cooking behaviour before and during the corona pandemic in the period from April 22th to 27th 2020 (Table 1). The questionnaire and the idea for this research was developed by an international consortia of universities and research institutions under the led of the Danish Technology Institute, and the Copenhagen Business School and can be found as supplementary information.^4^

**Table 1:** Sociodemography of the survey sample $(n=973)$

	%
Gender: female	42.7
Ages groups (years)	
20–39	31.5
40–59	38.8
60+	29.7
Household constellation	
With children (0–18 years)	23.1
With children (0–12 years)	12.0
Two adults without children	47.0
Singles	30.0
School education	
Low	10.5
Middle	54.1
High	35.5
Income loss: yes	26.1

The respondents were recruited via the consumer panel of the agency respondi.^5^ Responsibility for household shopping was used as a screening parameter. Only people responsible for purchasing groceries or who stated to share this task at least to 50% with other members of the household were allowed to participate. The collected data went through a quality and plausibility check by the German Institute of Food Technology’s consumer science research platform. The online questionnaire was sent to the panellists via the DIL - Quick Smart-Survey Server.^6^

To measure the change in the total amount of food consumed due to Covid-19, a five-point Likert scale with the categories “much less”, “slightly less”, “no change”, “a little more” and “much more” was applied. To measure the change across different product groups, respondents were asked how often they consumed these foods before and during the Covid-19 pandemic for each analyzed product group. The studied product groups were fruit/vegetables, meat, fish, bread, milk, frozen goods, canned food, ready-made meals, cakes/biscuits, sweets and alcohol. The response options for the consumption frequency ranged from “less than once every two weeks or never” to “daily” (Table 2).

**Table 2:** Answer options on the frequency of consumption of various foods

Coding	Answer categories
1	Less then once every 2 weeks or never
2	Between once a week and once each 2 weeks
3	Once a week
4	2–3 times per week
5	4–6 times per week
6	Daily

For the analysis for each product category (Fig. 3), the mean value (based on the number coding for the response categories) before the pandemic $({\bar{x}}_{before\ Covid\text{-}19})$ and the mean of the change caused by the pandemic $({\bar{x}}_{change}={\bar{x}}_{before\ Covid\text{-}19}-{\bar{x}}_{during\ Covid\text{-}19})$ were calculated. A two-sided *t*-test was applied to analyze whether the consumption frequency in a product category was significantly changed due to the corona pandemic. The ${H}_0$-hypothesis aimed to check whether the measured change in the consumption quantity was equal to zero $(\mu _{change}=0).$ The change in consumption frequency is also shown at an individual level in the result section. The values of the numerical coding for the consumption frequency before and during the pandemic were subtracted from each other at the level of the individual respondent (formula I), so that numbers of a maximum of +5 (change from “daily” to “less than once every two weeks or never”) to $-5$ (change from “Less than once every two weeks or never” to “Daily”) were possible results (Fig. 3):

1 $$\begin{aligned} \varDelta Eating\ freq.&=\,  {} {Eat.\ freq.}_{during\ Covid\text{-}19}\\ &\quad-\,{Eat.\ freq.}_{before\ Covid\text{-}19}. \end{aligned}$$

To measure further changes in consumer behaviour due to the corona pandemic, again a 5-pole Likert scale with the answer options “much less”, “a little less”, “no change”, “a little more” and “much more” was used. The $\chi ^2$-test was used to test the relationship between consumers’ fear of not getting food and the question of whether more food would be stored during the pandemic (Hair et al. 2010).

The study is relying on the hypothesis that the pandemic has a stronger impact in certain household segments (see Sect. 3.2). Therefore, four household segments were created for an in-depth analysis (Table 3). The presence of children in the household and the extent to which a corona-related loss of income was affected were used as segmentation variables. Differences in consumer behaviour of the analysed households segments were checked via a row of Fisher’s exact tests for $2\times 2$ tables. For this purpose the 5-pole Likert scale with the answer options “much less”, “a little less”, “no change”, “a little more” and “much more” was condensed into the three categories “less” (sum of “much less” and “little less’), “no change” and “more” (sum of “little more” and “much more”).

**Table 3:** Sample size of the analyzed household segments

	Total sample	No kids and no income loss	Kids and no income loss	No kids and income loss	Kids and income loss
Sample size	973	579	140	169	85
${\emptyset }$ Household size	2.29	1.82	4.04	1.79	3.61
Age (years)					
20–39	31.5	23.8%	46.4%	34.9%	52.9%
40–59	38.8	33.7%	47.1%	45.0%	47.1%
60+	29.7	42.5%	6.4%	20.1%	–
Education					
Low	10.5	12.4%	5.7%	9.5%	7.1%
Middle	54.1	53.7%	54.3%	52.1%	60.0%
High	35.5	33.9%	40.0%	38.5%	32.9%

## Results and discussion

### Stockpiling and the influence of risk perception

Almost a third of the respondents (31.4%) indicated to stockpile more food compared to the time before the pandemic. Simultaneously, the fear of not getting enough food was increased. Before Covid-19, very few study participants (3%) were anxious to this effect. In contrast, this value increased almost to 18% (sum of the values “often” and “occasionally”) at the time of the survey (Table 4). Concerning the influence of fear on not getting enough food and stockpiling, the results show that the bigger the fear, the more the study participants were stockpiling ($\chi ^2=55.164;$ *df* = 2, *p* < 0.001).

**Table 4:** Has anyone in your household been anxious about obtaining enough food to meet their requirements before and during Covid-19?

	Before Covid-19 in %	During Covid-19 in %
Frequently	0.2	1.5
Occasionally	2.7	16.2
Never	97.1	82.2

### Change in the amount of food consumed

**Fig. 1:**
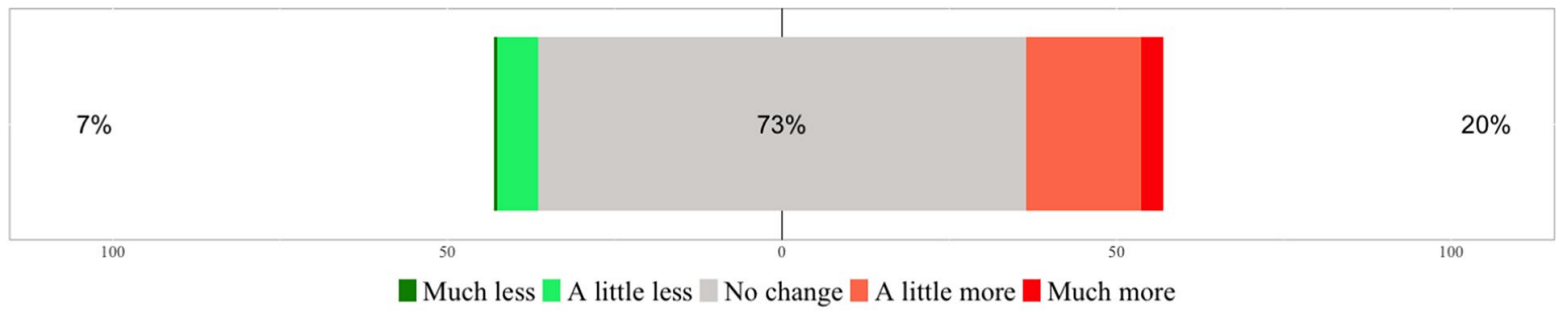
Change in the amount of food consumed during Covid-19

A central question of this study was, if people consumed more food during the pandemic. Across the entire sample, 20.5%, i.e. around a fifth of the respondents, stated that “more food” was consumed in their household (sum of the top values “much more” and “a little more”) (Fig. 1).

**Fig. 2:**
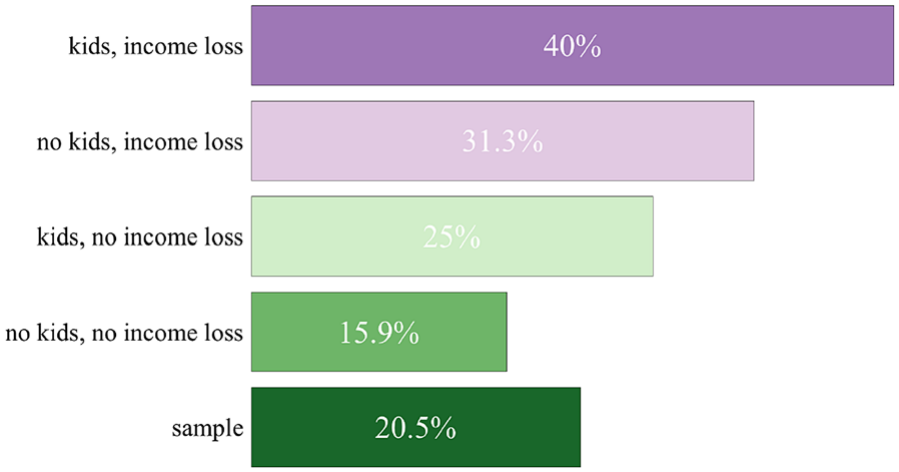
Share of households with an increased consumption of food overall in different household segments (top scores “much more” and “a little more”)

When the increased consumption was analyzed for the different household segments, a high degree of heterogeneity in the population became obvious (Fig. 2). In households with no children and no income loss, the increase was lower compared to the average. In contrast, there was an increased caloric intake, especially in households with children and/or pandemic-related income losses.

Based on fisher’s exact test for $2\times 2$ tables (Table 5) it was checked if the mentioned differences between different household segments were significant. This holds for most *p*-values which supports the finding that income loss and kids in the household are drivers for an increased food consumption during the pandemic.

**Table 5:** Fisher’s exact test—*p*-values for groups differences between households segments concerning the increased food consumption

	No kids, income loss vs. kids, income loss	No kids, income loss vs. kids, no income loss	No kids, income loss vs. no kids, no income loss
Less vs. no change	0.81	0.01	0.02
Less vs. more	0.15	0.05	0.87
No change vs. more	0.06	0.43	0.00

	Kids, income loss vs. kids, no income loss	Kids, income loss vs. no kids, no income loss	Kids, no income loss vs. no kids, no income loss
Less vs. no change	0.08	0.27	0.28
Less vs. more	0.74	0.07	0.02
No change vs. more	0.01	0.00	0.01

### Change in consumption frequency of various product categories

**Fig. 3:**
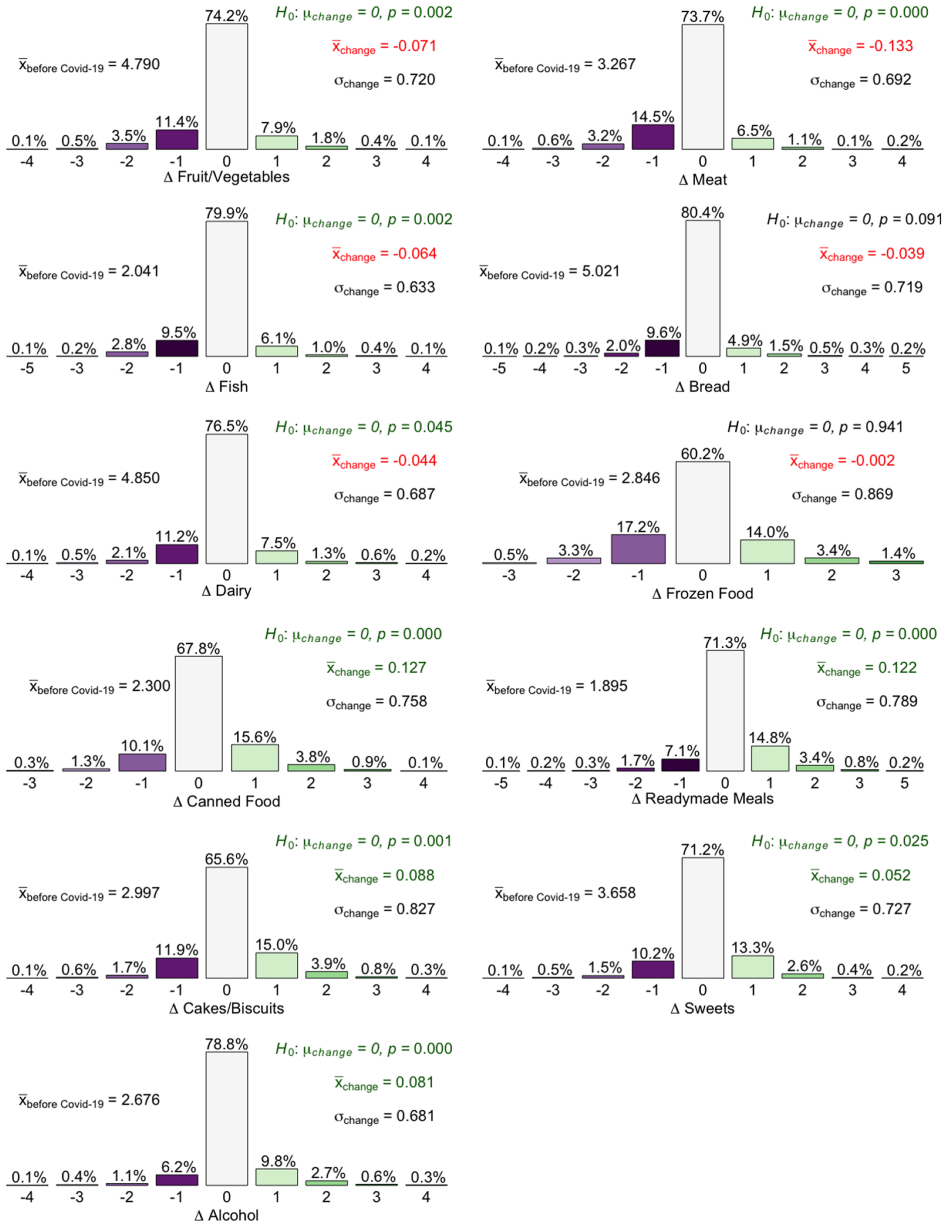
Change in consumption frequency in certain product categories

During the corona lockdown, there were significant decreases in the frequency of consumption of fruits/vegetables, fish and meat (Fig. 3). In contrast, there were significant increases in the categories of canned goods, ready-made meals, cakes/cookies, sweets and alcohol. Thus, there was a tendency for fresh products to be partly substituted by more processed, and more durable (convenience) products or partially unhealthy foods (sweets, alcohol). In the context of the measured risen overall food consumption, there was not only a substitution but an additional consumption of the latter mentioned products.

For the different household segments (Table 6), again large group differences become apparent. E.g., 13.4% of the respondents declared a higher consumption of alcohol. However, this parameter increased only to 11.5% for households without children and without loss of income. In contrast, an increased alcohol consumption is found more frequently (21.2%) in households with children and with reduced income.

For fruits and vegetables only 10.8% of the households with children and without income loss stated to consume less of these products. Compared to the time period before Covid-19, the consumption even increased during the pandemic. The opposite was true for households with children and a loss of income, (17.7%). In this group, the consumption of fruits and vegetables was on average reduced during lockdown.

Contrarily to fruits and vegetables, only 16.6% of respondents from households with children and with no loss of income reported an increased consumption of ready-made meals. For households with children and a loss of income, a much higher value could be found (28.3%). Similar results are shown for frozen food. In the product category meat, especially in households with children and a loss of income, meat consumption has declined (27.8%). In households with children without loss of income, this value was only 17.8%.

Based on fisher’s exact test for $2\times 2$ tables (Tables S8–14) it was checked if the mentioned differences between different household segments were significant. The analysis revealed that some but not the majority of the group differences were significant. But, it is to highlight that for two groups we had relatively small sample sizes. It is to expect that more of the analysed group differences are significant when a larger sample is considered.

**Table 6:** Change in consumption frequency according to product groups and household segments (top scores—“much more” and “a little more” respectively “a little less” and “much less”)

	Sample (%)	No kids and no income loss (%)	Kids and no income loss (%)	No kids and income loss (%)	Kids and income loss (%)
More alcohol	13.4	11.5	13.6	17.1	21.2
Less fruits/vegetables	15.5	13.2	10.8	16.7	17.7
More ready-meals	19.2	16.6	15.7	23.6	28.3
More tinned food	20.4	18.4	22.8	24.8	24.7
More frozen food	18.8	16.7	16.4	20.8	26.0
More sweets	16.9	15.0	17.1	17.8	22.4
Less meat	18.4	16.7	17.8	20.8	27.8

### Price perception

During the corona pandemic, there were price increases for meat and vegetables (Akter 2020). Agricultural economic research shows that for lower-income households the elasticity of the demand is in general stronger in comparison to other household segments (Thiele and Weiss 2003). That is plausible, because households with a lower income have to calculate more precisely to get along with their financial budget. Accordingly, the demand of higher-income households reacts less elastic. Based on this, it is to highlight that food prices during the corona pandemic were perceived very different across the segments. The majority (63.9%) of households with children and income losses stated to spent more money on food compared to the pre-Covid-19 period. For households with children but no loss of income, this value was only 25.0%.

### Changed eating habits in the context of the aspect of sustainability

**Fig. 4:**
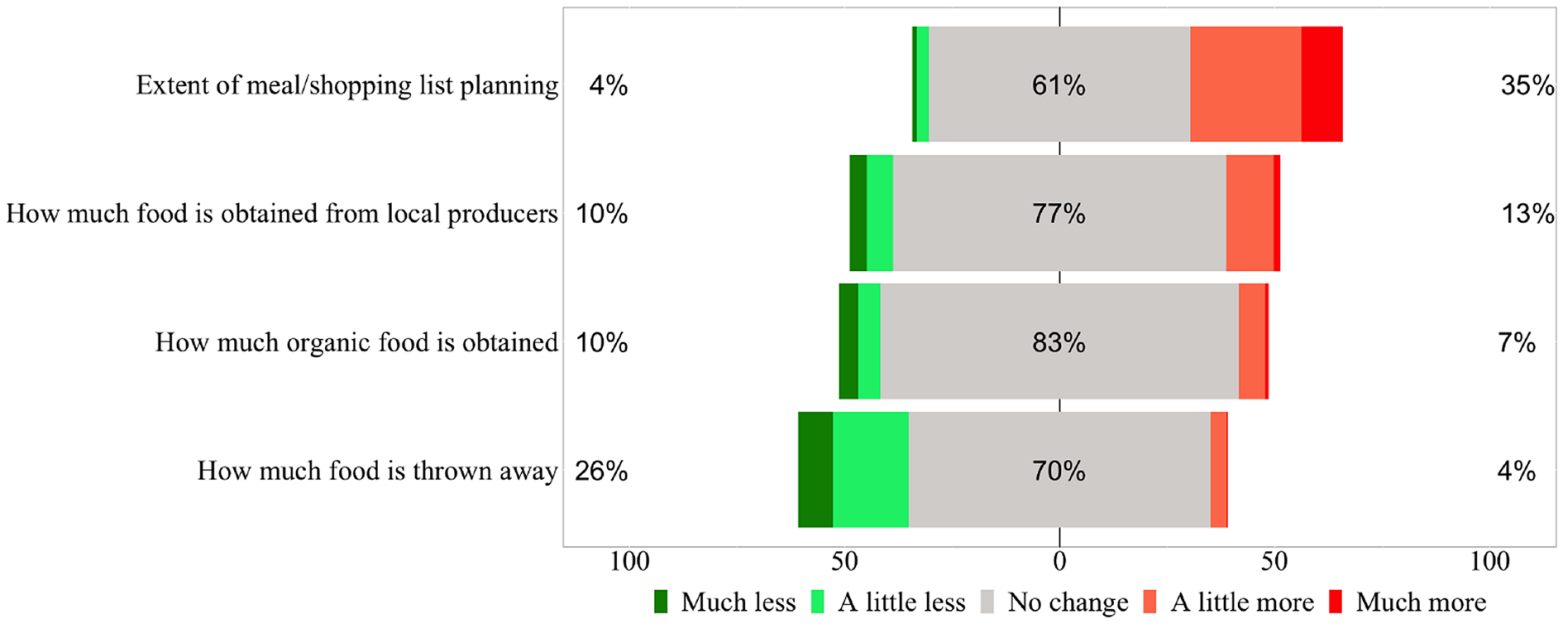
Change in sustainable household behavior due to the corona pandemic

The study revealed only minor changes in consumer’s behaviour concerning locally or organically produced food. There was no push towards locally or organically produced products as a result of the pandemic (Fig. 4). The changes in the positive as well as in the negative direction almost compensate each other.

In the context of sustainability, however, it can be shown that a relatively large group of 26% of the households threw away less food. In addition, more than a third planned meals and/or their grocery list more in advance. Based on a $\chi ^2$-square test, a significant relationship between the changed extent of planning and food waste avoidance could be found. Households, which planned “a lot more” or “more” in advance indicated more often to threw away “a little less” or “much less” food during the pandemic ($\chi ^2=139{,}77$; *df* = 16; *p* < 0.001).

Across household segments, differences in the change of sustainable consumer behaviour could be found. In particular in households with kids, meals and shopping lists are planned more in advance (Table 7). Concerning local food, the highest rise can be found for households with kids but no income loss. Likewise this group indicated compared to all other segment most often (30.0%) to produce less food waste during the pandemic. Interestingly, in households with kids but an income loss the lowest value for the reduction of food waste could be found (22.4%).

**Table 7:** Change in sustainable household behaviour according to product groups and household segments (top scores—“much more” and “a little more” respectively “a little less” and “much less”)

	Sample (%)	No kids and no income loss (%)	Kids and no income loss (%)	No kids and income loss (%)	Kids and income loss (%)
More planning	35.4	31.8	42.1	32.3	44.7
More local	12.5	11.9	17.1	10.7	12.9
More organic	7.0	6.9	6.4	7.1	8.2
Less food waste	25.8	24.2	30.0	29.6	22.4

## Conclusions

This empirical study demonstrates that the corona lockdown has influenced eating habits of households. More food was eaten, and more convenience products such as ready meals and canned food with a longer shelf life were purchased. The consumption of alcohol and sweets has also increased. In return, the consumption of fresh fruits and vegetables declined.

These changes occurred to varying degrees in different household types. The overall increase in food consumption could be measured in particular for households with children and corona-related income losses. In households affected by budget restrictions, the factor “child” alone leads to a deterioration in the household diet. In other words, an increase in calorie intake that is well above average and a stronger switch towards an increased consumption of more unhealthy product groups. In this context, it is worrying that alcohol consumption has risen most strongly in precisely these households. Families who are financially affected by the pandemic represent a vulnerable group. With the ongoing pandemic, repeated lockdowns, corona-related closings of schools and kindergartens, severe health consequences are expected long term, especially for this population group. The following measures can be taken by politicians and other stakeholders related to food production to counteract this negative development:

- Schools and kindergartens should be kept open. This could have a direct influence on the children’s nutrition through meal planning. The application of the DGE quality standard^7^ for healthy and sustainable catering in community facilities for children should be made mandatory in this context.
- The quality of community catering for children must increase. Freshness, health and enjoyment should be in the foreground, and the caloric content of the menus adapted according to children’s age. In addition, constant random quality controls by higher-level authorities are required.
- The municipalities and districts with the support of the state governments should not act according to the standard “good and above all cheap”.

Due to the increase in the consumption of more processed food, the industry can also contribute to improve the health value of these (convenience) products by using more gentle and improved production methods and processes (e.g. high-pressure technology, pulsed electric fields). Government support can be provided from two sides in this context: By focussing on sustainable and healthy food in research funding to optimize the underlying procedures and processes or, if necessary, to develop them. Second, we propose to set the Nutri-Score as mandatory by law. The Nutri-Score is using a Nutrient Profiling System (NPS) to define five different categories of nutritional quality (from dark green associated with the letter A to dark orange with an E). The letter A represents a preferable score and E a detrimental score. Recent research demonstrates that the classification of foods according to the Nutri-Score is consistent with German Food-Based Dietary Guidelines. Food consumption as recommended by the guidelines is more favourably classified (e.g. most products composed mainly of fruits and vegetables were classified as A or B) than foods which consumption should be limited (e.g. nearly all of sugary snacks were classified as D or E) (Szabo de Edelenyi et al. 2019). Therefore, the Nutri-Score allows the consumer to differentiate between the offered options and to identify healthy alternatives. This holds in particular for more processed convenience goods as ready-made meals. Dréano-Trécant et al. (2020) found for composite dishes a wide distribution concerning the Nutri-Score, which highlighted the large variability in prepared products in terms of nutritional quality, for which the Nutri-Score is a very useful tool to identify healthier options.”

It is to highlight that a considerable part of the population feared not getting enough food during the lockdown. Therefore, we recommend that responsible state ministries run educational campaigns in non-pandemic times to encourage the population in stockpiling certain amount of foods on a mid- to long-term basis in order to avoid a run on grocery stores in times of crisis. Nonetheless, there is need for research to what extent this measure can prevent out-of-stock situations. Furthermore, it should be analysed to what extent the communication of political actors in the media has triggered the fear of not getting enough food.

## Supplementary information

Below is the link to the electronic supplementary material.

Supplementary file3 (pdf 642 KB)

## Data Availability

The data presented in this study are available on request from the corresponding author. The data will be made publicly to a later stage.

## Footnotes

1 http://www.li-food.de, Accessed on 08.07.2021.

2 https://innofact-marktforschung.de/news/innofact-corona-studie-wer-hamstert-eigentlich-die-nudeln/, Accessed on 11.08.2021., Accessed on 08.07.2021.

3 http://www.arbeitsagentur.de, Accessed on 08.07.2021.

4 http://www.food-covid-19.org, Accessed on 08.07.2021.

5 http://www.respondi.com, Accessed on 08.07.2021.

6 http://www.survey.dil-ev.de, Accessed on 08.07.2021.

7 http://www.schuleplusessen.de, Accessed on 08.07.2021.
